# Cytokine levels is septic shock

**DOI:** 10.1186/2197-425X-3-S1-A296

**Published:** 2015-10-01

**Authors:** L Mateu Campos, M Arlandis Tomas, L Galarza Barrachina, F Sanchez Morán, A Belenguer Muncharaz, D Ferrándiz Sellés, B Vidal Tegedor, F Bernal Julián, E Rodríguez Martínez

**Affiliations:** Hospital General de Castellon, Castellón de la Plana, Spain; Hospital de la Plana, Vila-Real, Spain

## Objectives

Evaluate the profile of cytokines levels in a group of septic patients and the relationship with organ dysfunction.

## Methods

Patients admitted to the ICU with criteria of septic shock (SCCC 2001), all of them mechanically ventilated and requiring vasoactive support. Demographic variables, renal function and haemodynamic monitoring data by PiCCOÓ system were collected. Tumor necrosis factor (TNF-a), interleukin (IL) 6, IL-8, IL-10 levels, IL-10/ TNF-a ratio and lactate were analyzed at admission (T1), at 48 (T2) and 72 hours (T3), and at discharge from ICU or prior to death (T4). Ten patients with anemia served as controls for determination of cytokine levels.

## Results

Twenty-three patients were included (mean age 74.5 years; 52% males patients). The type of admission was surgical in 11 patients and medical in 12 patients. APACHE II score was 18.33 ± 6.59 and SOFA score at admission 10.8 ± 2.58. The overall mortality of the group was 9 patients.On admission, patients with septic shock had higher levels of all cytokines than did controls, with significant differences for IL-6 and IL-8 (p < 0.05).There were significant differences in baseline serum levels of IL-10 and IL-10 /TNF-a ratio between survivors and non-survivors, and these differences remained at 48 hours. The levels of IL-6 were higher in non-surviving group at 48 hours and at the end of the study (p < 0.05). A significant correlation of baseline IL-10 /TNF-a ratio with SOFA score was noted. In addition, IL-8 significantly correlated with plasma creatinine (p < 0.05) and a statistically significant negative correlation was obtained between the cardiac index with the levels of IL-6, IL-8 and TNF-a. All of cytokines revealed a statistically significant positive correlation with lactate levels.

## Conclusions

Cytokine levels had a different progression between patients. Sustained intravascular inflammation characterized by a continuous elevation of pro-inflammatory cytokines as well as a predominance of anti-inflammatory profile determines which patients have a worse final outcome. The pro-inflammatory cytokines are involved in myocardial dysfunction associated to sepsis, as well as renal dysfunction.

